# Incidental screen positive findings in a prospective cohort study in Matlab, Bangladesh: insights into expanded newborn screening for low-resource settings

**DOI:** 10.1186/s13023-018-0993-1

**Published:** 2019-01-30

**Authors:** Malia S. Q. Murphy, Pranesh Chakraborty, Jesmin Pervin, Anisur Rahman, Lindsay A. Wilson, Monica Lamoureux, Kathryn Denize, Matthew Henderson, Steve Hawken, Beth K. Potter, Julian Little, Kumanan Wilson

**Affiliations:** 1 Clinical Epidemiology Program, Ottawa Hospital Research Institute, 1053 Carling Ave, Ottawa, K1Y 4E9 Canada; 2 International Centre for Diarrhoeal Disease Research, Dhaka, Bangladesh; 3 Newborn Screening Ontario, Children’s Hospital of Eastern Ontario, 401 Smyth Rd, Ottawa, K1H 5B2 Canada; 4 School of Epidemiology and Public Health, University of Ottawa, 600 Peter Morand Crescent, Ottawa, K1G 5Z3 Canada

**Keywords:** Newborn screening, Congenital hypothyroidism, Hemoglobin disorders, Congenital disorders

## Abstract

**Background:**

Newborn screening programs are essential preventative public health initiatives but are not widely available in low-resource settings. The objective of this study was to describe the frequency and nature of screen positive determinations as made by a Canadian newborn screening program in a cohort of infants born in Matlab, Bangladesh. Dried newborn cord and heel-prick blood spot samples collected as part of a validation study nested within a preterm birth research cohort were collected between January 2017 and July 2018 and analyzed in a Canadian newborn screening laboratory where the laboratory’s disease panel and screening thresholds were applied.

**Results:**

A total of 1661 newborn samples (520 heel-prick and 1141 cord blood samples) were available for analysis. Based on the applied screening thresholds, 61 samples (22 by heel-prick and 39 by cord blood) were screen positive for conditions included in the Canadian disease panel. Congenital hypothyroidism was the most common determination for heel-prick (*n* = 17) and cord blood (*n* = 12) samples. Carriers of hemoglobinopathy variants were identified in 6.9% of both tested heel-prick and cord blood samples.

**Conclusions:**

This study provides insight into the nature and frequency of treatable congenital conditions in a rural Bangladesh community where such data were previously unavailable. As comment to the feasibility of newborn screening in the region we confirm that screening based on cord blood sampling continues to be the most acceptable modality to parents in such settings. Acknowledged barriers include early infant discharge, which may affect the reliability of initial screening thresholds to determine disease risk. We further highlight the importance of continuing efforts in the country to identify infants with congenital hypothyroidism.

**Electronic supplementary material:**

The online version of this article (10.1186/s13023-018-0993-1) contains supplementary material, which is available to authorized users.

## Background

Newborn screening programs are essential preventative public health initiatives designed to provide timely identification of infants with rare but treatable conditions. Screening encompasses laboratory testing, retrieval of screen positive infants, diagnostic testing, genetic counselling services and ongoing care of affected infants. Although privately- and publicly-funded programs are available throughout much of Western Europe and North America, many economic, political and cultural barriers exist to the successful implementation and long-term management of newborn screening programs in developing countries in the Middle East, North Africa and the Asia Pacific region [1]. Emerging screening initiatives are typically established as private programs, and their institutionalization within the health care system is dependent on government support and recognition. Those supported by academic or hospital initiatives have historically exhibited slow progress and growth [1].

Screening efforts in Bangladesh, as in other low- and middle-income countries, have focused on popularizing the program and building the necessary infrastructure to ensure long-term success [2, 3]. Bangladesh is one of the most densely populated countries in the world, with a population of over 160 million and over 2 million infants born per year [4, 2]. Over 70% of the population live in rural areas, whereas most of the tertiary-level health care centres are located in the areas surrounding Dhaka [5]. Although pilot newborn screening for congenital hypothyroidism (CH) was implemented in 1999 using in-country laboratories and resources [6], single disease screening has been limited to < 5% of infants [1]. The research collaboration between a large Canadian newborn screening program and a team conducting a birth cohort study in Bangladesh, aimed at determining the utility of newborn screening analytes for establishing preterm birth rates [7], presented an unprecedented opportunity to examine data generated from use of an expanded newborn screening disease panel in this population. Our objective was to describe the frequency and nature of screen positive results for congenital, treatable conditions in this cohort of Bangladeshi infants.

## Results

### Participant demographics

A total of 1661 newborn samples (520 heel-prick and 1141 cord blood) were collected between 1 January 2017 and 31 July 2018. A total of 1178 infants contributed samples, with 658 having cord samples only, 37 having heel samples only and 483 having both sample types. Preterm infants comprised 6.7 and 9.5% of the heel-prick and cord blood sample cohorts, respectively. Overall, the majority of samples were derived from infants who were ≥ 2500 g at birth (81.7% heel-prick; 83.8% cord blood). All cord blood samples and the majority of heel-prick samples were collected within 24 h of birth. Timing of sample collection did not differ between preterm infants and term infants. Newborn characteristics are summarized in Table 1.

**Table 1:** Summary of patient characteristics

	Heel Samples *N* = 520	Cord Samples *N* = 1141
Sex		
Male, n(%)	258 (49.6%)	578 (50.7%)
Female, n(%)	262 (50.4%)	563 (49.3%)
Completed weeks of gestational age, wks (SD)	39.2 (1.5)	39.0 (1.6)
≥ 37 weeks, n(%)	485 (93.3%)	1033 (90.5%)
32–36 weeks, n(%)	34 (6.5%)	105 (9.2%)
28–31 weeks, n(%)	1 (0.2%)	2 (0.2%)
< 28 weeks, n(%)	0 (0.0%)	1 (0.1%)
Birthweight, g (SD)	2834.7 (429.4)	2859.5 (439.6)
≥ 4000 g, n(%)	3 (0.6%)	15 (1.3%)
2500 g to < 4000 g, n(%)	422 (81.2%)	941 (82.5%)
1500 g to < 2500 g, n(%)	91 (17.5%)	177 (15.5%)
1000 g to < 1500 g, n(%)	4 (0.8%)	4 (0.4%)
< 1000 g, n(%)	0 (0.00%)	3 (0.3%)
Multiple birth, n(%)	7 (1.3%)	19 (1.7%)
Newborn age at sample collection, hrs (SD)	16.2 (8.5)	0.08 (0.07)
Term infants (SD)	16.3 (8.5)	0.08 (0.07)
Preterm infants (SD)	16.2 (8.8)	0.08 (0.07)
< 24 h	467 (89.8%)	1141 (100.0%)
24–72 h	53 (10.2%)	0 (0.0%)
> 72 h	0 (0.0%)	0 (0.0%)

Data are presented as mean (standard deviation) or n (%) as indicated

### Screen positive determinations

**Fig. 1:**
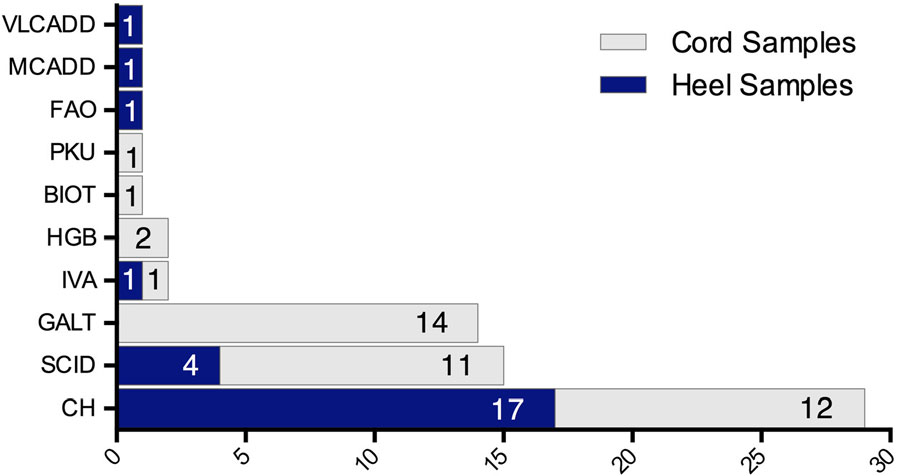
Frequency of screen positive determinations by sample type. CH, congenital hypothyroidism; SCID, severe combined immunodeficiency; GALT, galactosemia; IVA, isovaleric academia; HGB, hemoglobinopathy; BIOT, biotinidase deficiency; PKU, phenylketonuria; FAO, untargetted fatty acid oxidation disease; MCADD, medium chain acyl coA dehydrogenase deficiency; VLADD, very long chain acyl coA dehydrogenase deficiency

The frequency of screen positive determinations based on standard thresholds applied by the newborn screening laboratory are presented in Fig. 1. Screen positive determinations included those for amino acidemias (phenylketonuria), organic acidemias (isovaleric acidemia), fatty acid oxidation defects (medium chain acyl co-A dehydrogenase deficiency, very long chain acyl co-A dehydrogenase deficiency and others), galactosemia, biotinidase deficiency, hemoglobinopathies and severe combined immune deficiency. CH was the most common screen positive determination overall. Of the 1661 newborn samples analyzed over the course of this study, 61 samples from 60 unique infants were screen positive. Five infants received screen positive flags for two or more conditions. 33 of the 60 infants with screen positive determinations had both heel-prick and cord blood samples analyzed. Only one infant received screen positive determinations in both sample types, but the screen positive determinations did not match.

Application of study-specific screening thresholds to manage incidental findings of CH yielded one screen positive determination (TSH > 50mLU/L; diagnosis was confirmed, and treatment initiated). No study-specific actionable results for MCADD or hemoglobinopathies were identified.

### Thyroid stimulating hormone and congenital hypothyroidism

TSH assays were run on 516 heel-prick samples and 1126 cord blood samples. Overall, TSH levels from heel-prick samples (mean 6.64mLU/L ± SD 4.43mLU/L) were higher than levels taken from cord blood (mean 3.73mLU/L ± SD 10.58mLU/L). 17 (3.3%) of 516 infants screened by heel-prick and 12 (1.1%) of 1126 infants screened by cord blood were screen positive for CH based on screening thresholds set in Ontario, Canada. Screen positive CH infants had TSH levels ranging from 17.1–29.2mLU/L and 17.0–337.4mLU/L for heel-prick and cord blood, respectively.

**Fig. 2:**
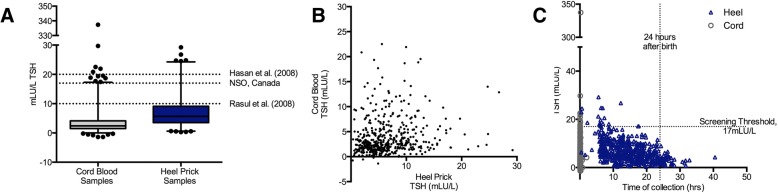
Distribution of TSH levels by sample type. TSH levels for 516 infants screened by heel-prick and 1126 infants screened by cord blood. **a** TSH levels were 6.64 ± 4.43mLU/L for heel-prick samples, and 3.73 ± 10.58mLU/L cord blood levels (mean ± SD). Screening thresholds to identify infants requiring follow-up used in Ontario, Canada (17 mLU/L) and previously reported for pilot CH screening programs in Bangladesh (20 mLU/L, Hasan et al. 2008 [2]; and 10 mLU/L, Rasul et al. 2009 [9]) are indicated. **b** Correlation of TSH levels measured in 476 paired heel-prick and cord blood samples, Spearman *R* = 0.22 (95%CI 0.13–0.31). **c** Distribution of TSH values by timing of sample collection. TSH values below zero represent those that were below the detection limit of the assay

Paired TSH levels for heel-prick and cord blood samples were obtained from 476 infants. Among this subgroup, TSH levels were also higher in heel-prick samples (mean 6.62mLU/L ± SD 4.42mLU/L) than in cord blood samples (mean 3.91mLU/L ± SD 3.78mLU/L). Twenty-three of the screen-positive determinations for CH were made in infants who had both cord and heel-prick samples analyzed, although in all cases, analysis of the corresponding sample yielded screen-negative findings. There was a trend toward decreasing TSH levels with later sample collection. All screen positive determinations of CH identified in the course of this study (TSH > 18mLU/L) were determined from samples collected within the first 24 h of life. Figure 2 provides the distribution of TSH levels for cord blood and heel-prick samples overall and by newborn age, and the correlation of TSH levels between paired samples.

### Hemoglobin variants, carrier status and Hemoglobinopathies

Hemoglobin peak percentage data were available from 520 (100.0%) heel-prick samples and 1132 (99.2%) cord blood samples (Table 2). The majority of samples were obtained from screen negative infants who were non-carriers for any hemoglobinopathies (93.1% heel-prick; 92.7% cord blood). HbE carriers were identified in 5.9% of heel-prick samples and 6.4% of cord blood samples. HbD carriers and other carrier traits constituted < 1% of the samples tested. Two infants were screen positive for hemoglobinopathies, although no cases of sickle cell diseases or thalassemia major were identified. Thus, no study-specific actionable screening results were generated.

**Table 2:** Distribution of hemoglobin variants

	Heel, n = 520	Cord, *n* = 1132
Negative, non-carrier	484 (93.1%)	1049 (92.7%)
Carrier HbD	2 (0.4%)	2 (0.2%)
Carrier HbE	31 (5.9%)	72 (6.4%)
Carrier Other	3 (0.6%)	4 (0.4%)
Sickle/Beta-Thalassemia, HB-FEa	–	1 (0.1%)
HB-Other Positive	–	1 (0.1%)
Unavailable; unsatisfactory sample quality	–	3 (0.3%)

## Discussion

In this study, newborn blood spot samples collected for a research study in Matlab, Bangladesh underwent analysis at a newborn screening lab in Ontario, Canada. We have used this opportunity to describe the nature and frequency of screen positive determinations made by an expanded newborn screening program in Canada among infants born in a rural Bangladesh community where such a service is not currently available. Analysis of over 1600 cord blood and newborn heel-prick samples yielded 67 screen positive determinations in 60 unique infants based on Canadian screening thresholds. CH was the most common screen positive determination made in both sample types.

Pilot initiatives in Bangladesh to date has been focused on screening for CH [6], a common cause of irreversible cognitive disability when left untreated. Global incidence of CH is reportedly 1:3000–1:4000, subject to regional variation and availability of newborn screening. Bangladesh is a known area of endemic iodine deficiency where the incidence of CH is expected to be high, although the issue has not been systematically studied [6, 8]. Cord blood screening of 31,802 randomly selected infants from 10 hospitals located primarily in Dhaka City from 2000 to 2006 identified 438 (1.4%) infants with TSH > 20mLU/L. 362 of the screen positive cases (82.6%) were successfully recalled for confirmatory testing [2], and 16 cases of CH confirmed (1:2000). In a separate region of the country, the Khulna District in southern Bangladesh, 35 of 1353 infants (2.6%) were positive for CH by cord blood dried blood spot analysis based on an initial screening threshold of 10 mLU/L TSH [9]. As in Dhaka, not all recalled families returned for confirmatory testing (68.5%); diagnosis was confirmed in two infants (1.5 per 1000). In our own study, application of Canadian newborn screening procedures and thresholds to newborn samples from Bangladesh indicated a similar screen positive rate to those previously reported by these pilot programs. 1.9% of infants screened by cord blood would have been recalled if screening thresholds used in Ontario, Canada were applied (intermediary threshold of 17mLU/L relative to previously reported thresholds). However, the high sampling rate before 24 h of age in our cohort, including among preterm infants should be noted. For these reasons, discordant CH screening results for the twenty-three samples with paired cord and heel-prick data are unsurprising. In Ontario, any sample taken within 24 h of birth are considered ‘unsatisfactory’, and a repeat sample requested. High rates of discharge within 24 h of birth and limited opportunities to re-sample in many low-resource settings may thus affect the reliability of initial screening tests to determine disease risk.

Samples in our study were also used to examine hemoglobin variants. Bangladesh is located in a region of the world with a relatively high prevalence of thalassemia and other hereditary hemoglobin disorders are also not uncommon [5, 10–12]. Available literature suggests particularly high rates of beta thalassemia and HbE in Bangladesh, particularly amongst the country’s tribal populations [13, 14]. HbE carriers comprise approximately 6% of the population in Bangladesh [12, 13], a rate consistent with the findings of our study (6.8%). Given the prevalence of thalassemias and other hemoglobinopathies in this region of the world, there may be benefit in leveraging newborn blood spots collected for CH for hemoglobinopathy screening. Unlike the more technically demanding tandem mass spectrometry platforms required for metabolic screening, identification of hemoglobin variants may be made by HPLC or gel electrophoresis. Analyses may also be reliable from cord blood samples.

Although newborn screening efforts in Bangladesh are generally well-accepted by government and health officials, the literature commonly reports problems related to sample quality control, and insufficient resources to support adequate training and staffing early in the program [9, 3], a matter confirmed by our own experience. Recognized socio-cultural barriers, including the high rate of at-home births (~ 80%), early infant discharge and lack of parental education about newborn screening are also major barriers to implementing a sustainable screening program in Bangladesh and similar countries [1, 15]. Cord blood is subject to maternal admixture and requires rapid collection after delivery, but permits sample collection where mother-infant pairs are discharged more rapidly, is logistically easier to collect and has better parental acceptance than heel-prick samples [9, 3]. It is for these reasons that emerging CH screening programs often include cord blood collection [16, 17], despite the fact that TSH may be more reliably measured by heel-prick sampling 1–2 days after birth.

We note that our data were derived in the course of a non-interventional research project. Due to the nature of this study, screening cutoffs were not customized beyond the clinically relevant/high PPV concept, and diagnostic data are unavailable. While management of incidental findings included case-reporting for CH; MCADD; and hemoglobinopathies [7], only initial testing values were available. Repeat and diagnostic testing were performed locally in Matlab. As a result, we are unable to speak to the true-positive rates of the screened conditions based on standard reporting thresholds used by the screening laboratory. Although the frequency of screen positive cases defined by this study’s screening thresholds may appear high, we highlight that for the majority of these conditions, the PPVs are relatively low [18, 19]. High false-positive rates are particularly expected when testing samples collected within 24 h after birth in the case of CH screening [18]. As noted above, the Ontario provincial screening lab considers samples taken within 24 h to be ‘unsatisfactory’. False positive rates are also high for samples taken from infants born preterm and low birthweight for both CH and SCID [20, 21]. Specifically, all 26 screen-positive samples for CH were collected earlier than 24 h after birth (cord or heel-prick based analyses), 2(7.7%) were preterm and 7(26.9%) had a birthweight < 2500 g. Additionally, six out of 13 infants (46.2%) who screened positive for SCID were preterm, and 7(53.8%) were low birthweight. Although we did not identify any screen positives for congenital adrenal hyperplasia, the potential for false positives in samples taken before 24 h also exists with this condition. Finally, we acknowledge the limitation of using cord blood for interpretation of newborn screening findings but again highlight the better acceptance of this sample type by parents, the feasibility of its collection, and its use for newborn screening by others.

Selection of conditions to be included in local or national newborn screening programs for low-income countries is challenging. Programs in high-income countries rely on established policies and criteria to guide considerations of whether disorders should be added to existing programs. Based on well-established principles for public health screening [22, 23], these criteria typically include significant risk of illness, disability or death without early treatment; existence of effective treatments that are widely accessible; presence of programs and policies to deal with diagnostic testing, counselling, treatment and follow-up; and newborn benefits, and whether these factors are reasonably balanced against financial and other costs [24–26]. Cost-reduction in newborn screening has historically been addressed through limiting the size of the disease panel, adjusting recall thresholds, and use of privately-funded programs. The cost-effectiveness of CH screening in particular is recognized in developed settings where estimated lifetime costs of untreated CH cases are estimated to be between $USD1–2 million [27]. Analyses in developing countries suggest similar benefits where sustainable programs are emerging [28, 29]. However, in low-resource settings such as Bangladesh, where health systems are stressed by competing health burdens, scalability of newborn screening is impeded. Screen positive rates for CH in this analysis based on thresholds used by a newborn screening program in Ontario, Canada would suggest that in a country with roughly 163 million people and a birthrate of 18.8/1000, 57,000 infants would be recalled for additional testing per year if a national newborn screening program were available. Applying the PPV of CH screening in Ontario of 54.7% [18] at current screening thresholds suggests that 31,179 infants might be true positives; but the PPV would likely be higher in Bangladesh given the higher rates of iodine deficiency disorders. Adjusted PPVs provide a strategy to offset costs associated with newborn screening. While this might result in missed opportunities to treat, recalling only those infants for whom there is a near certainty of CH status may be an economically favourable alternative for programs relying on government and external financial support.

## Conclusions

This study provides insight into the frequency and nature of congenital conditions in a rural community where newborn screening is not offered. Where newborn screening is recognized as an essential public health program, much work is needed to develop the means to support screening, retrieval of screen positive patients, and ensure proper diagnosis and follow-up care of infants in low-resource nations.

## Methods

### Aim

The aim of this study was to describe the frequency and nature of screen positive findings for an expanded newborn screening disease panel in a cohort of Bangladeshi infants.

### Study design and setting

Data for this study were derived from a prospective study nested within ‘Preterm and Stillbirth Study, Matlab’ (PreSSMat); a preterm birth cohort study designed to capture data on the biological determinants of preterm birth. Pregnant women enrolled in the PreSSMat cohort were followed prospectively throughout pregnancy and at 6-weeks post-partum to collect demographic, socioeconomic, nutritional and physical data as well as maternal and neonatal biological specimens. PreSSMat was conducted in the Matlab sub-district of Chandpur, Bangladesh, where the women of reproductive age and children under 5 years of age living in the icddr,b service area receive care though icddr,b facilities. This study was approved by the Ottawa Health Science Network Research Ethics Board at The Ottawa Hospital Research Institute (20160219-01H) and the Research Ethics Board of the Children’s Hospital of Eastern Ontario Research Institute (16/20E). The protocol was also approved by Ethical Review Committee at the icddr,b (PR-16039). All research participants provided written informed consent.

### Participants

Participant recruitment and sample collection were performed as previously described [7]. Written consent was obtained from parents of participating newborns in addition to the consent to participate in the PreSSMat cohort. Parents were informed of the nature of the study, the risks and benefits of participation, and how clinically actionable findings related to newborn screening would be handled. In brief, newborn blood samples were collected from both the umbilical cord and via newborn heel-prick. Parents could consent to the collection of one or both sample types from participating infants. There were no explicit exclusion criteria applied to the study cohort. Samples were collected 24–72 h after birth or prior to newborn discharge (whichever happened first), air-dried and stored in a temperature-controlled environment (21 °C) out of direct sunlight. Samples were sent on a weekly basis by international priority shipment to Ottawa, Canada for analysis.

We conducted an extensive training and quality assurance program from 1 August 2016 to 31 December 2017, prior to study initiation. This initiative included provision of training materials to clinical research staff for the collection, storage and shipment of newborn samples (*n* = 323). Samples were shipped to Ottawa, Canada where the physical quality of the samples were reviewed. Timing and logistics of sample storage and shipment were also considered. Quality feedback reports were provided to research staff in Matlab, Bangladesh on a per-shipment basis to optimize sample collection and shipment procedures. Quality assurance was halted when laboratory personnel were confident that Matlab staff could reliably collect and provide newborn screening specimens that could be used for clinical screening. Quality reporting continued throughout the duration of the research study.

### Newborn screening analysis

Samples were analyzed at Newborn Screening Ontario (NSO) as previously described [7]. NSO is a publicly-funded provincial newborn screening program located at the Children’s Hospital of Eastern Ontario in Ottawa, Canada that provides services to the approximately 140,000 infants born in Ontario each year. The NSO panel of analytes evaluated in each newborn dried blood sample includes over 50 initial markers to screen for treatable metabolic and endocrine conditions, sickle cell disease, cystic fibrosis and severe combined immune deficiency (Table 3).

**Table 3:** Conditions screened

Organic Acid Disorders	
Cobalamin A & B Defects	
Isovaleric Acidemia (IVA)	
Glutaric Acidemia Type 1 (GA1)	
Methylmalonic Acidemia (MMA)	
Propionic Acidemia (PA)	
Fatty Acid Oxidation Disorders	
Carnitine Uptake Defect (CUD)	
Fatty Acid Oxidation Disorders, other	
Medium Chain Acyl CoA Dehydrogenase Deficiency (MCADD)	
Long Chain 3-Hydroxyacyl-CoA Dehydrogenase Deficiency (LCHAD)	
Trifunctional Protein Deficiency (TFP)	
Very Long Chain Acyl CoA Dehydrogenase Deficiency (VLCAD)	
Amino Acid and Urea Cycle Disorders	
Phenylketonuria (PKU)	
Maple Syrup Urine Disease (MSUD)	
Homocystinuria	
Citrullinemia / Argininosuccinic aciduria	
Tyrosinemia Type 1	
Amino Acidopathies, other	
Other Metabolic Diseases	
Biotinidase Deficiency	
Galactosemia	
Endocrine Disorders	
Congenital Hypothyroidism (CH)	
Congenital Adrenal Hyperplasia (CAH)	
Other Genetic Diseases	
Cystic Fibrosis (CF)	
Hemoglobinopathies	
Sickle Cell Disease (Hemoglobin SC)	
Sickle Cell Disease (Hemoglobin SS)	
Sickle Cell Disease (Sickle/Beta-Thalassemia)	
Immune Deficiencies	
Severe Combined Immune Deficiency (SCID)	

The NSO analyte panel was run on each sample, provided there was sufficient sample for analysis. Where there was insufficient sample, samples underwent a partial analysis in adherence with a predefined analysis workflow. Screening algorithms and cutoffs used by NSO was applied to both heel-prick and cord blood samples to distinguish screen positive from screen negative cases. Although the data for this analysis are derived from a non-interventional cohort study, protocols were in place to guide the management of screen positive findings. In collaboration with PreSSMat investigators and the icddr,b ethics board, we prioritized the clinical reporting of conditions likely to have a higher birth prevalence in Bangladesh and where treatments were accessible and cost-efficient, using thresholds adjusted to increase certainty of screen positive status. On this basis, screen positive cases of CH, medium chain acyl co-A dehydrogenase deficiency (MCADD), and sickle cell diseases were deemed clinically actionable. The screening thresholds for CH and MCADD were adjusted to achieve PPVs of > 99% in the Bangladesh cohort [18, 19]. Screening cut-offs were not customized beyond the clinically relevant/high PPV concept. By standard, screening samples in Ontario are considered ‘unsatisfactory’ if taken when the infant is less than 24 h of age, and a repeat sample requested. Samples in this study were collected 24–72 h after birth or prior to discharge if the newborn was released from hospital within 24 h of delivery.

Reports for screen positive cases of these ‘high-priority’ conditions were generated and provided to the investigation team in Matlab, and clinical follow-up facilitated. Clinical care programmes were at the discretion of the Matlab team and local health care providers. Diagnostic data and reports generated by health care providers in Matlab were not available to the study team, although confirmation of diagnostic results and initiation of disease management were relayed. Screen positive determinations for all other conditions identified by the NSO newborn screening panel were provided to the Matlab team on a quarterly basis. The details of the instrumentation and screening algorithms applied to all samples are provided in the Additional file 1.

## Additional file

Additional file 1: Supplementary materials and methods. (DOCX 79 kb)
