# Supplementary material for: Incidental screen positive findings in a prospective cohort study in Matlab, Bangladesh: insights into expanded newborn screening for low-resource settings
Source: Orphanet J Rare Dis. 2019 Jan 30;14:25. doi: 10.1186/s13023-018-0993-1 (PMC6354381; doi:10.1186/s13023-018-0993-1)
Supplement: Supplementary file 1 — Supplementary materials and methods. (DOCX 79 kb) [file 13023_2018_993_MOESM1_ESM.docx]

**Additional file 1: Supplementary Materials and Methods**

**Laboratory Instrumentation**

Hemoglobin profiles were determined by high performance liquid chromatography on a Bio Rad Variant^TM^ nbs system. Neonatal 17-hydroxyprogesterone (17-OHP), thyroid stimulating hormone (TSH) and immunoreactive trypsinogen (IRT) were measured using a PerkinElmer AutoDELFIA® Immunoassays. Amino acid and acylcarnitine analysis was performed by electrospray ionization tandem mass spectrometry (Waters TQ Detector). Total TREC copy number was measured by quantitative polymerase chain reaction using a ThermoFisher Scientific Viia 7. Biotinidase and galactose-1-phosphate uridyltransferase levels were measured using the Astoria-Pacific SPOTCHECK® Pro system.

**Screening Algorithms**

The following ‘final phase’ screening algorithms was applied to both heel prick and cord blood samples. Determination of ‘Screen Positive’ was made accordingly. Further to the screening logic provided below, CHEO-RI provided real-time Alert reports for ‘Screen Positive’ cases of Congenital Hypothyroidism (CH), Medium Chain Acyl Co-A Dehydrogenase Deficiency (MCADD) and Sickle Cell Disease using the following criteria.

- MCADD: C8>1 AND C8/C10>6 (100%PPV)
- CH: thyroid stimulating hormone>50 (99%PPV)
- Sickle Cell Disease: FS, FSE, or FSC hemoglobin patterns. Also F only, in keeping with beta-thalassemia major.

If there was an insufficient quantity of blood to fulfill a complete analysis, a priority punching protocol was a applied and a partial analysis compelted. The priority punching was as follows:

1. Amino acids and Acyl Carnitines (AAAC)
2. Hemoglobin Profiles
3. Thyroid stimulating hormone
4. 17 hydroxy progesterone
5. Biotinidase
6. Galactosemia
7. T-cell receptor excision circles
8. Immunoreactive trypsinogen

For cord sample analyses, second tier steroid profiling was not completed.

|  | **INITIAL PHASE** | | | | | **FINAL PHASE** | | | | | |
| --- | --- | --- | --- | --- | --- | --- | --- | --- | --- | --- | --- |
|  | **ANALYTES** | | IMPRECISION LOGIC | | ALERT LOGIC | **ANALYTES** | | | | **CONFIRMATION LOGIC** | |
|  | Primary | Secondary | < 7 DAYS OLD | >7 DAY OLD | ALL AGES | Subsequent Tier(s) | Primary | Secondary | Others to include on Risk Letter | < 7 DAY OLD | > 7 DAY OLD |
| ***Fatty Acid Oxidation Defects*** | | | | | | | | | | | |
| MCAD | C8 | C8/C2 | C8**≥0.5** OR [C8≥**0.4** AND (C6≥0.25 OR C10:1≥0.15 OR C10≥0.30 OR C8/C2≥0.01 OR C8/C10≥2.50)] | C8>0.40 | (C8>1 AND C8/C10>6) |  | C8 | C8/C2 | C8 | C8**≥0.5** OR [C8≥**0.4** AND (C6≥0.25 OR C10:1≥0.15 OR C10≥0.30 OR C8/C2≥0.01 OR C8/C10≥2.50)] | C8≥0.40 |
|  |  | C8/C10 |  |  |  |  |  | C8/C10 | C5DC_C10OH |  |  |
|  |  | C6 |  |  |  |  |  | C6 | C12 |  |  |
|  |  | C10 |  |  |  |  |  | C10 | C14:1 |  |  |
|  |  | C10:1 |  |  |  |  |  | C10:1 |  |  |  |
| LCHAD  **OR MTP** | C16OH | C14OH | C16OH**≥**0.20 OR [C16OH≥0.15 AND (C14OH≥0.18 OR C16:1OH≥0.15 OR C18OH≥0.10 OR C16OH/C16≥0.086) | C16OH>0.17 | C16OH>0.5 |  | C16OH | C14OH | C2 | C16OH≥0.20 OR [C16OH≥0.15 AND (C14OH≥0.18 OR C16:1OH≥0.15 OR C18OH≥0.10 OR OR C16OH/C16≥0.086) | C16OH≥0.17 |
|  |  | C16:1OH |  |  |  |  |  | C16:1OH | C14 |  |  |
|  |  | C18OH |  |  |  |  |  | C18OH | C14:1 |  |  |
|  |  | C16OH/C16 |  |  |  |  |  | C16OH/C16 | C16 |  |  |
|  |  |  |  |  |  |  |  |  | C18 |  |  |
|  |  |  |  |  |  |  |  |  | C18:1 |  |  |
| VLCAD | C14:1 | C12:1 | C14:1**≥**0.65 OR [C14:1≥0.37 AND (C12:1≥0.50 OR C14≥0.70 OR C14:2≥0.15 OR C14:1/C12:1≥5.00 OR C14:1/C16≥0.203 OR C14:1/C4≥4.85)] | C14:1>0.40 | C14:1>1 AND C14:1/C16>0.75 |  | C14:1 | C12:1 | **C2** | C14:1≥0.65 OR [C14:1≥0.37 AND (C12:1≥0.50 OR C14≥0.70 OR C14:2≥0.15 OR C14:1/C12:1≥5.00 OR C14:1/C16≥203 OR C14:1/C4≥4.85)] | C14:1≥0.40 |
|  |  | C14 |  |  |  |  |  | C14 | C16 |  |  |
|  |  | C14:2 |  |  |  |  |  | C14:2 | C18 |  |  |
|  |  | C14:1/C16 |  |  |  |  |  | C14:1/C16 | C18:1 |  |  |
|  |  | C14:1/C4 |  |  |  |  |  | C14:1/C4 |  |  |  |
| CUD | C0 | (C0+C2+C3+C16+C18) / Cit | C0<9.5 OR [C0<11.5 AND (C16<1.5 OR C18<0.05 OR (C0+C2+C3+C16+C18)/Cit<3.00)] | n/a |  |  | C0 | (C0+C2+C3+C16+C18) / Cit | Cit | C0<9.5 OR [C0<11.5 AND (C16<1.5 OR C18<0.05 OR (C0+C2+C3+C16+C18)/Cit<3.00)] | n/a |
|  |  | C16 |  |  |  |  |  | C16 | C2 |  |  |
|  |  | C18 |  |  |  |  |  | C18 | C3 |  |  |
|  |  |  |  |  |  |  |  |  | C18:1 |  |  |
|  |  |  |  |  |  |  |  |  | Met |  |  |
| FAO  (CPT1) | C0/  (C16+C18) |  | C0(C16+C18)≥30.00 AND (C0≥110.0 OR C16<1.5) |  |  | P479L mutation | C0/  (C16+C18) |  | C16 | C0(C16+C18)≥30.00 AND (C0≥110.0 OR C16<1.5) |  |
|  | C0 |  |  |  |  |  | C0 |  | C18 |  |  |
|  |  |  |  |  |  |  |  |  | C14 |  |  |
|  |  |  |  |  |  |  |  |  | C18:1 |  |  |
|  |  |  |  |  |  |  |  |  | C2 |  |  |
|  |  |  |  |  |  |  |  |  | C3 |  |  |
|  |  |  |  |  |  |  |  |  | C18:2 |  |  |
|  |  |  |  |  |  |  |  |  | P479L mutation |  |  |

|  | **INITIAL PHASE** | | | | | **FINAL PHASE** | | | | | |
| --- | --- | --- | --- | --- | --- | --- | --- | --- | --- | --- | --- |
|  | **ANALYTES** | | IMPRECISION LOGIC | | ALERT LOGIC | **ANALYTES** | | | | **CONFIRMATION LOGIC** | |
|  | Primary | Secondary | < 7 DAYS OLD | >7 DAY OLD | ALL AGES | Subsequent Tier(s) | Primary | Secondary | Others to include on Risk Letter | < 7 DAY OLD | > 7 DAY OLD |
| ***Organic Acidemias*** | | | | | | | | | | | |
| Propionic / Methylmalonic Acidemias (PA/MMA) | C3/C2 |  | C3/C2 ≥0.21 AND C3≥4.00 OR C3/C2 ≥0.23 And C3≥3.50 | C3/C2 ≥0.21 AND C3≥2.6 OR C3/C2 ≥0.23 AND C3≥2.4 | (C3/C2≥0.3 AND C3≥9) | MCA | C3/C2 | C3 | C3/C0 | [C3/C2≥0.23 AND MCA≥0.5)]  OR MCA≥0.7 | [C3/C2≥0.23 AND MCA≥0.5)]  OR MCA≥0.7 |
|  | C3 |  |  |  |  |  |  |  | C3/C4DC |  |  |
|  |  |  |  |  |  |  |  |  | C16:1OH/C4DC |  |  |
|  |  |  |  |  |  |  |  |  | C3/C16 |  |  |
| Isovaleric Acidemia / 2 Methylbutyric Acidemia (IVA) | C5 | C5/C2 | C5≥0.67 OR [C5≥0.60 AND (C5/C0≥0.03 OR C5/C2≥0.031 OR C5/C3≥0.50 OR C5/C4≥4.00 OR C5/C16≥0.70)] | C5>0.60 | (C5>1 AND C5/C2>0.25) |  | C5 | C5/C2 | C0 | C5≥0.67 OR [C5≥0.60 AND (C5/C0≥0.03 OR C5/C2≥0.031 OR C5/C3≥0.50 OR C5/C4≥4.00 OR C5/C16≥0.70)] | C5≥0.60 |
|  |  | C5/C0 |  |  |  |  |  | C5/C0 | C2 |  |  |
|  |  | C5/C3 |  |  |  |  |  | C5/C3 | C3 |  |  |
|  |  | C5/C4 |  |  |  |  |  |  | Met |  |  |
|  |  | C5/C16 |  |  |  |  |  |  | Val |  |  |
|  |  |  |  |  |  |  |  |  | Phe |  |  |
|  |  |  |  |  |  |  |  |  | Leu-Ileu (Xle) |  |  |
|  |  |  |  |  |  |  |  |  | C8:1 |  |  |
| Glutaric Acidemia Type 1 (GA1) | C5DC | C5DC/C16 | C5DC≥0.37 OR [C5DC≥0.31 AND (C5DC/C2≥0.01 OR C5DC/C16≥0.05 OR C5DC/C50H≥0.94 OR C5DC/C8≥1.25)] | C5DC≥0.31 |  |  | C5DC | C5DC/C16 | C5DC/C5OH | C5DC≥0.37 OR [C5DC≥0.31 AND (C5DC/C2≥0.01 OR C5DC/C16≥0.05 OR C5DC/C50H≥0.94 OR C5DC/C8≥1.25)] | C5DC≥0.31 |
|  |  | C5DC/C8 |  |  |  |  |  | C5DC/C8 | C8 |  |  |
|  |  | C5DC/C2 |  |  |  |  |  | C5DC/C2 | C6 |  |  |
|  |  | C5DC/C5OH |  |  |  |  |  | C5DC/C5OH | C10 |  |  |
|  |  |  |  |  |  |  |  |  | C10:1 |  |  |
|  |  |  |  |  |  |  |  |  | C12 |  |  |
|  |  |  |  |  |  |  |  |  | C14:1 |  |  |
|  |  |  |  |  |  |  |  |  | C16 |  |  |
|  |  |  |  |  |  |  |  |  | C18 |  |  |
|  |  |  |  |  |  |  |  |  | C18:1 |  |  |
|  |  |  |  |  |  |  |  |  | C5OH |  |  |
|  |  |  |  |  |  |  |  |  | C0 |  |  |
|  |  |  |  |  |  |  |  |  | C3DC |  |  |

|  | **INITIAL PHASE** | | | | | **FINAL PHASE** | | | | | |
| --- | --- | --- | --- | --- | --- | --- | --- | --- | --- | --- | --- |
|  | **ANALYTES** | | IMPRECISION LOGIC | | ALERT LOGIC | **ANALYTES** | | | | **CONFIRMATION LOGIC** | |
|  | Primary | Secondary | < 7 DAYS OLD | >7 DAY OLD | ALL AGES | Subsequent Tier(s) | Primary | Secondary | Others to include on Risk Letter | < 7 DAY OLD | > 7 DAY OLD |
| ***Amino Acidemias*** | | | | | | | | | | | |
| Maple Syrup Urine Disease (MSUD) | LEU |  | (LEU/ALA≥0.85 **AND** LEU≥250) |  | (LEU/ALA≥1.25 **AND** LEU≥300) |  | LEU |  | Valine | (LEU/ALA≥1.0 **AND** LEU≥300) |  |
|  | LEU/ALA |  |  |  |  |  | LEU/ALA |  | Alanine |  |  |
|  |  |  |  |  |  |  |  |  | Leu/Phe |  |  |
|  |  |  |  |  |  |  |  |  | Val/Phe |  |  |
|  |  |  |  |  |  |  |  |  | Met |  |  |
|  |  |  |  |  |  |  |  |  | Phe |  |  |
|  |  |  |  |  |  |  |  |  | Tyr |  |  |
| Phenylketonuria and Variants / Biopterin defects NBS-PKU (PKU) | PHE | PHE/TYR | PHE≥160 OR [PHE≥130 AND (PHE/TYR≥1.72)] |  |  |  | PHE | PHE/TYR | Tyrosine | PHE≥160 OR [PHE≥130 AND (PHE/TYR≥1.72)] |  |
|  |  |  |  |  |  |  |  |  | Met |  |  |
|  |  |  |  |  |  |  |  |  | Cit |  |  |
|  |  |  |  |  |  |  |  |  | Val |  |  |
|  |  |  |  |  |  |  |  |  | Leu-Ileu (Xle) |  |  |
| Tyrosinemia | SUAC |  | SUAC≥4.0 OR TYR≥600 |  | SUAC≥7.0 |  | SUAC |  | Phenylalanine | SUAC≥5.0 OR TYR≥600 |  |
|  | TYR |  |  |  |  |  | TYR |  | Methionine |  |  |
|  |  |  |  |  |  |  |  |  | Val |  |  |
|  |  |  |  |  |  |  |  |  | Leu-Ileu (Xle) |  |  |
| Homocystinuria | MET | MET/PHE | MET≥100 OR [MET≥47.0 AND (MET/PHE≥1.34)] |  |  |  | MET | MET/PHE | Phe | MET>100 OR [MET≥47.0 AND (MET/PHE≥1.34)] |  |
|  |  |  |  |  |  |  |  |  | Cit |  |  |
|  |  |  |  |  |  |  |  |  | Tyr |  |  |
|  |  |  |  |  |  |  |  |  | C3 |  |  |
|  |  |  |  |  |  |  |  |  | Leu-Ileu (Xle) |  |  |
| ***CITRULLINEMIAS*** | |  |  |  |  |  |  |  |  |  |  |
| Citrullinemia | CIT | CIT/ARG | CIT≥70 OR [CIT≥40 AND (ASA≥2.5 OR CIT/ARG≥6.61 OR CIT/ORN≥2.40 OR ASA/ORN≥0.10 OR ASA/ARG≥0.12)] |  | CIT≥100 | daugters of 459 | CIT | CIT/ARG | Arginine | {CIT≥70 OR [CIT≥40 AND (ASA≥2.5 OR CIT/ARG≥6.61 OR CIT/ORN≥2.40 OR ASA/ORN≥0.10 OR ASA/ARG≥0.12]} AND Normal daughters of 459 |  |
|  |  | ASA |  |  |  |  |  | ASA | Cit/Arg |  |  |
|  |  | CIT/ORN |  |  |  |  |  | CIT/ORN | Orn |  |  |
|  |  | ASA/ARG |  |  |  |  |  | ASA/ARG | Ala |  |  |
|  |  |  |  |  |  |  |  |  | Met |  |  |
|  |  |  |  |  |  |  |  |  | Phe |  |  |
| Argininosuccinic Aciduria | CIT | CIT/ARG |  |  |  | daugters of 459 | CIT | CIT/ARG | Arginine | {CIT≥70 OR [CIT≥40 AND (ASA≥2.5 OR CIT/ARG≥6.61 OR CIT/ORN≥2.40 OR ASA/ORN≥0.10 OR ASA/ARG≥0.12]} AND ABNormal daughters of 459 |  |
|  |  | ASA |  |  |  |  |  | ASA | Cit/Arg |  |  |
|  |  | CIT/ORN |  |  |  |  |  | CIT/ORN | Orn |  |  |
|  |  | ASA/ARG |  |  |  |  |  | ASA/ARG | Ala |  |  |
|  |  |  |  |  |  |  |  |  | Met |  |  |
|  |  |  |  |  |  |  |  |  | Phe |  |  |

|  | **INITIAL PHASE** | | | | | **FINAL PHASE** | | | | | |
| --- | --- | --- | --- | --- | --- | --- | --- | --- | --- | --- | --- |
|  | **ANALYTES** | | IMPRECISION LOGIC | | ALERT LOGIC | **ANALYTES** | | | | **CONFIRMATION LOGIC** | |
|  | Primary | Secondary | < 7 DAYS OLD | >7 DAY OLD | ALL AGES | Subsequent Tier(s) | Primary | Secondary | Others to include on Risk Letter | < 7 DAY OLD | > 7 DAY OLD |
| ***Other*** | | | | | | | | | | | |
| Galactosemia (GALT) | GALT |  | GALT<2 |  | GALT≤1.5 |  | GALT |  |  | GALT**≤**1.5 |  |
| Biotinidase Deficiency (BIOT) | BIOT |  | BIOT**<40.0** |  |  |  | BIOT |  |  | BIOT**≤**27.0 |  |
| ***IMMUNOLOGY*** | | | | | | | | | | | |
| SCID | TREC |  | TREC < 75 |  |  | TREC2 |  |  | Valine | TREC2 < 75 OR  [dAdo≥1.0 OR Ado≥5.9 OR Gua≥17.5 OR dGua≥8.2) OR (TREC2<75 and TBX1Rq <0.6) |  |
|  |  |  |  |  |  | TBX1 |  |  | Alanine |  |  |
|  |  |  |  |  |  | Adenosine |  |  | Leu/Phe |  |  |
|  |  |  |  |  |  | Deoxy-adenosine |  |  | Val/Phe |  |  |
|  |  |  |  |  |  | Guanosine |  |  | Met |  |  |
|  |  |  |  |  |  | Deoxy-guanosine |  |  | Phe |  |  |
|  |  |  |  |  |  | RNAseP |  |  | Tyr |  |  |
| ***ENDOCRINOLOGY*** | | | | | | | | | | | |
| Congenital Hypothyroidism (CH) | TSH |  | TSH**≥**13 |  |  |  | TSH |  |  | TSH**≥**17 |  |
| Congenital Adrenal Hyperplasia (CAH) | 17OHP |  | (BW≥2500 AND 17OHP**≥**38.0)  OR (BW 1500-2499 AND 17OHP**≥**52.0) OR (BW 1000-1499 AND 17OHP**≥**91.0) OR (BW <1000 AND 17OHP**≥**160.0) | For samples collected at > 21 days of age, the most conservative cutoff (i.e.38) applies,   regardless of the birth weight |  | 17OHP-MS |  |  |  | 17OHP-MS**≥**15.0 AND [(17OHP**+4A)/C**]**≥**0.4 |  |
|  | BW |  |  |  |  | Cortisol-MS |  |  |  |  |  |
|  |  |  |  |  |  | Androstenedioene-MS |  |  |  |  |  |
|  |  |  |  |  |  |  |  |  |  |  |  |
|  |  |  |  |  |  |  |  |  |  |  |  |
|  |  |  |  |  |  |  |  |  |  |  |  |
|  |  |  |  |  |  |  |  |  |  |  |  |
| ***CYSTIC FIBROSIS*** | | | | | | | | | | | |
| Cystic Fibrosis (CF) | IRT |  | IRT>Assay 96th centile OR IRT>monthly minimum cutoff |  |  | CF mutations |  |  |  | [IRT>Assay 96th centile OR IRT>monthly minimum cutoff) AND 1 mutation] OR 2 mutations OR IRT>99.9th centile |  |
|  |  |  |  |  |  |  |  |  |  |  |  |
|  |  |  |  |  |  |  |  |  |  |  |  |
|  |  |  |  |  |  |  |  |  |  |  |  |
|  |  |  |  |  |  |  |  |  |  |  |  |
|  |  |  |  |  |  |  |  |  |  |  |  |
| ***HEMATOLOGY*** | | | | | | | | | | | |
| Sickle Cell Disease (SCD) | Hb profile |  | FS, FSa, FSC, FSD, FSE, FSX, F, FC, FD, FE, FX, FCa, FDa, FEa, FXa, FCX, FDX, FEX, FCE, FCD, FDE, Other (Positive) |  |  |  | Hb profile |  |  | FS, FSa, FSC, FSD, FSE, FSX, F, FC, FD, FE, FX, FCa, FDa, FEa, FXa, FCX, FDX, FEX, FCE, FCD, FDE, Other (Positive) |  |
|  |  |  |  |  |  |  |  |  |  |  |  |
|  |  |  |  |  |  |  |  |  |  |  |  |
|  |  |  |  |  |  |  |  |  |  |  |  |
